# Comparison of pharmacological inhibitors of lysine-specific demethylase 1 in glioblastoma stem cells reveals inhibitor-specific efficacy profiles

**DOI:** 10.3389/fneur.2023.1112207

**Published:** 2023-04-04

**Authors:** Lea M. Stitzlein, Achintyan Gangadharan, Leslie M. Walsh, Deokhwa Nam, Alexsandra B. Espejo, Melissa M. Singh, Kareena H. Patel, Yue Lu, Xiaoping Su, Ravesanker Ezhilarasan, Joy Gumin, Sanjay Singh, Erik Sulman, Frederick F. Lang, Joya Chandra

**Affiliations:** ^1^Department of Pediatrics Research, University of Texas MD Anderson Cancer Center, Houston, TX, United States; ^2^The University of Texas MD Anderson Cancer Center UTHealth Graduate School of Biomedical Sciences, Houston, TX, United States; ^3^Department of Epigenetics and Molecular Carcinogenesis, University of Texas MD Anderson Cancer Center, Houston, TX, United States; ^4^Department of Bioinformatics and Computational Biology, University of Texas MD Anderson Cancer Center, Houston, TX, United States; ^5^Department of Radiation Oncology, NYU Langone Medical Center, New York, NY, United States; ^6^Department of Neurosurgery, University of Texas MD Anderson Cancer Center, Houston, TX, United States

**Keywords:** histone demethylase inhibitors, epigenetics, lysine specific demethylase (KDM), glioblastoma, drug resistance

## Abstract

**Introduction:**

Improved therapies for glioblastoma (GBM) are desperately needed and require preclinical evaluation in models that capture tumor heterogeneity and intrinsic resistance seen in patients. Epigenetic alterations have been well documented in GBM and lysine-specific demethylase 1 (LSD1/KDM1A) is amongst the chromatin modifiers implicated in stem cell maintenance, growth and differentiation. Pharmacological inhibition of LSD1 is clinically relevant, with numerous compounds in various phases of preclinical and clinical development, but an evaluation and comparison of LSD1 inhibitors in patient-derived GBM models is lacking.

**Methods:**

To assess concordance between knockdown of LSD1 and inhibition of LSD1 using a prototype inhibitor in GBM, we performed RNA-seq to identify genes and biological processes associated with inhibition. Efficacy of various LSD1 inhibitors was assessed in nine patient-derived glioblastoma stem cell (GSC) lines and an orthotopic xenograft mouse model.

**Results:**

LSD1 inhibitors had cytotoxic and selective effects regardless of GSC radiosensitivity or molecular subtype. In vivo, LSD1 inhibition via GSK-LSD1 led to a delayed reduction in tumor burden; however, tumor regrowth occurred. Comparison of GBM lines by RNA-seq was used to identify genes that may predict resistance to LSD1 inhibitors. We identified five genes that correlate with resistance to LSD1 inhibition in treatment resistant GSCs, in GSK-LSD1 treated mice, and in GBM patients with low LSD1 expression.

**Conclusion:**

Collectively, the growth inhibitory effects of LSD1 inhibition across a panel of GSC models and identification of genes that may predict resistance has potential to guide future combination therapies.

## 1. Introduction

Prognosis for patients with glioblastoma (GBM) is poor with an average survival of 12–15 months even with first-line therapy (1–3). Targeted therapy for molecularly defined subsets of GBM has been tested extensively but is largely met with drug resistance and minimal improvements in survival, prompting the need to develop more effective treatment options. Resistance to targeted therapies is in part attributed to heterogeneity across the tumor. Existence of such intratumoral heterogeneity is thought to be maintained by the presence of a tumor subpopulation called glioblastoma stem cells (GSCs) (4–7). GSCs have stem cell-like properties, including self-renewal, that are key to repopulating a highly adaptive tumor that can evade treatment efficacy (4, 6). The presence of GSCs in GBM are maintained by frequent mutations and a permissive epigenetic landscape (6, 8). Therefore, focusing on dysregulated chromatin modulators in GBM may directly suppress GSCs and prompt a sustained treatment response.

One epigenetic mediator of stemness in GSCs is lysine-specific demethylase 1 (LSD1/KDM1A) (9). In GBM, LSD1 has demonstrated a role in maintaining GSC stemness and promoting tumorgenicity (8, 10) and the repression of LSD1 is associated with induction of differentiation, apoptosis, and loss of stemness in GSCs (8, 11). Given the role of LSD1 in GSC maintenance, it is an attractive target to reshape the epigenetic landscape in GBM and facilitate a robust response to treatment.

Targeting LSD1 in GBM can be accomplished with pharmacological inhibitors. The first identified LSD1 inhibitor was tranylcypromine (TCP), a brain penetrant drug approved to treat anxiety disorders. Accordingly, TCP served as a lead molecule that led to the synthesis of several more selective LSD1 inhibitors. TCP and the successive compounds are irreversible, catalytic inhibitors of LSD1. Additionally, the scaffolding properties of LSD1 and its ability to associate with regulatory complexes can be inhibited (12). Overall, an evaluation of the more specific pharmacological LSD1 inhibitors and their effects on GSCs is lacking.

In the present study we compare the transcriptomic profile of GBM cells following LSD1 knockdown with LSD1 inhibition to define genes that are relevant to pharmacological inhibition. We then used nine patient-derived MDA-GSC lines to evaluate the *in vitro* sensitivity and selectivity of seven LSD1 inhibitors, GSK-LSD1, TCP, RN-1, SP-2577, ORY-1001, ORY-2001, and IMG-7289. We report differential responses on MDA-GSC viability to the seven LSD1 inhibitors that are irrespective of sensitivity to radiation, or GBM molecular subtype, indicating potential for efficacy across a range of GBM categories. Guided by our *in vitro* data, we assessed the *in vivo* effects of LSD1 inhibition in orthotopic xenograft models of MDA-GSCs and observed a significant drop in tumor burden after long-term treatment with the LSD1 inhibitor, GSK-LSD1, which was transient with tumor regrowth occurring. To understand the molecular basis for this tumor response, the effects of LSD1 inhibition on gene expression were assessed using RNA-Seq and we established a set of genes that correlate with resistance to LSD1 inhibition. These genes were examined *in vitro* from treatment resistant MDA-GSC lines and *in vivo* following LSD1 inhibition treatment. Lastly, we confirmed the presence of the genes in RNA sequencing data from glioblastoma patients acquired from TCGA where they showed an inverse relationship with LSD1 expression. Collectively, these data highlight unique effects of LSD1 inhibitors in MDA-GSC models and identify genes that may predict resistance and could be targeted in future combination strategies.

## 2. Materials and methods

### 2.1. Reagents

GSK-LSD1 (CAS #2102933–95-7), SP-2577 (CAS #1423715–37-0), and IMG-7289 (CAS #1990504–72-7) were provided by GlaxoSmithKline, Salarius Pharmaceuticals, and Imago BioSciences, respectively through materials transfer agreements. TCP and RN-1 were purchased through Enzo Life Sciences (Farmingdale, NY) and Cayman Chemical (Ann Arbor, MI). ORY-1001 and ORY-2001 were purchased through Cayman Chemical (Ann Arbor, MI) and MedChemExpress (Monmouth Junction, NJ). All LSD1 inhibitors were diluted per manufacturer’s instructions, aliquoted into single-use vials, and stored at −80°C. Table 1 illustrates the chemical structures for LSD1 inhibitors utilized in this study.

**Table 1:** Chemical structure of LSD1 inhibitors evaluated in the GSC models.

LSD1 inhibitor	Structure
GSK-LSD1	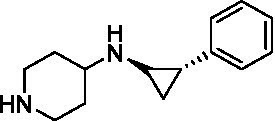
SP-2577	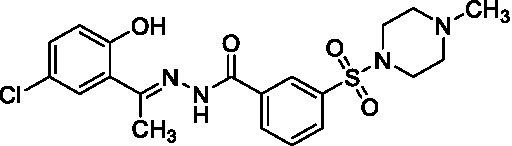
IMG-7289	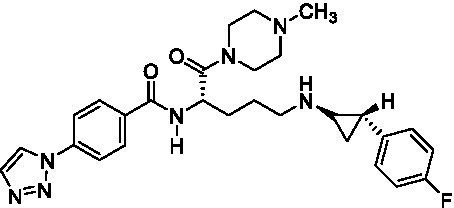
Tranylcypromine	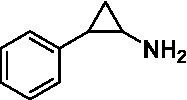
RN-1	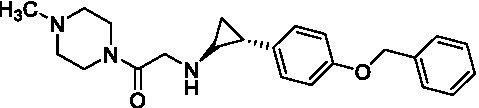
ORY-1001	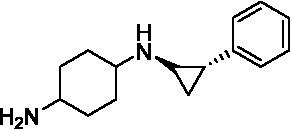
ORY-2001	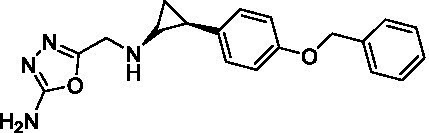

### 2.2. Cell lines

Human MDA-GSC lines (MDA-GSC262, MDA-GSC311, MDA-GSC20, MDA-GSC17, MDA-GSC6-27, MDA-GSC7-11, MDA-GSC23, MDA-GSC8-11, MDA-GSC7-2) were derived from patients with GBM and established with informed consent after approval from the University of Texas MD Anderson Institutional Review Board under protocol LAB 04–0001 led by Dr. Lang as previously described (13–15). This panel of MDA-GSC lines was molecularly characterized by the Brain Tumor Center at MD Anderson Cancer Center and was chosen for further studies as it represents a broad range of GBM categories with differences in sensitivity to radiation, molecular classification, and genomic landscape (Table 2). Sensitivity to radiation was determined *in vitro* by evaluating survival fraction of MDA-GSCs post-radiation (0, 2, 4, and 6Gy) compared to non-irradiated controls. The MDA-GSCs are also characterized as primary or recurrent GBM (Table 3).

**Table 2:** Molecular classification, radiation sensitivity characterization, and mutation status of the MDA-GSC panel.

	Molecular classification	Sensitivity to radiation	MGMT status	Mutations in cancer related genes					
				*TP53*	*PTEN*	*PIK3CA*	*NF1*	*FANCD2*	*RB1*
MDA-GSC262	Proneural	Resistant	Methylated	–	–	–	–	–	–
MDA-GSC311	Unavailable	Unavailable	Unavailable	–	–	–	–	–	–
MDA-GSC20	Mesenchymal	Resistant	Methylated	–	–	–	–	–	–
MDA-GSC17	Proneural	Sensitive	Unmethylated	Yes	Yes	No	No	No	No
MDA-GSC6-27	Classical	Unavailable	Unmethylated	No	No	Yes	Yes	No	No
MDA-GSC7-11	Proneural	Resistant	Methylated	Yes	Yes	No	No	No	No
MDA-GSC23	Classical	Resistant	Unmethylated	–	–	–	–	–	–
MDA-GSC8-11	Proneural	Sensitive	Methylated	Yes	No	No	No	Yes	Yes
MDA-GSC7-2	Classical	Sensitive	Methylated	–	–	–	–	–	–

— Mutation status not determined for the given cell line.

**Table 3:** MDA-GSC lines characteristics at the time of establishment.

	Diagnosis	Treatment
MDA-GSC262	Primary GBM	None
MDA-GSC311	Primary GBM	None
MDA-GSC20	Primary GBM	None
MDA-GSC17	Recurrent GBM	Patient underwent radiotherapy and TMZ and was completed in 1/2005. Patient was then treated with a dose intense TMZ. GSC line generated in 8/2005 after recurrence.
MDA-GSC6-27	Primary GBM	None
MDA-GSC7-11	Primary GBM	None
MDA-GSC23	Primary GBM	None
MDA-GSC8-11	Primary GBM	None
MDA-GSC7-2	Primary GBM	None

TMZ - temozolomide.

Immortalized normal human astrocytes (NHA/E6/E7/Tert) were purchased through Lonza (Basel, Switzerland), while the LN18 were purchased at ATCC (Manassas, VA). The LNZ308 cell line was provided by Dr. Oliver Bolger. The MDA-GSCs were grown in T75 suspension culture flasks (Genesee Scientific; San Diego, CA) as neurospheres and maintained in DMEM/F-12 50/50 (Corning; Corning, NY) with 2 mM L-glutamine (Gibco; Waltham, MA), and 100 units/ml-100 ug/mL penicillin–streptomycin solution (GeminiBio; West Sacramento, CA). On the day of culturing the base media was supplemented with 2% B-27 (Invitrogen; Waltham, MA), EGF 20 ng/ml (Shenandoah Biotechnology), FGF 20 ng/ml (Shenandoah Biotechnology; Warminster, PA), and 0.0002% heparin solution (StemCell Technologies; Vancouver, Canada). The MDA-GSC20 luciferase labeled cells (MDA-GSC20-luc) were transfected with a firefly luciferase reporter and confirmed to be bioluminescent using D-luciferin (GoldBio; St. Louis, MO) on a luminescent plate reader. The LN18, LNZ308, and NHA cells were grown as an adherent monolayer in T75 flasks (BioBasic; Ontario, Canada) using DMEM/F-12 50/50 with 10% fetal bovine serum (Hyclone Laboratories; Logan, UT), 2 mM L-glutamine, and 100 units/ml-100 μg/mL penicillin–streptomycin solution. NHA were also cultured with selection antibiotics puromycin 0.5 μg/mL and blasticidin S 10 ug/mL (Cayman Chemical; Ann Arbor, MI). All cell lines were grown at 37°C in a humidified atmosphere containing 5% CO_2_. The LN18 LSD1 knockdown cell line was achieved with shRNA construct with the sequence GGCGAAGGTAGAGTACAGAGA. Transfection proceeded using the nucleofector technology following manufacturer’s protocol with program T-20.

### 2.3. RNA-Seq analysis

Total RNA was extracted using the RNeasy Plus Mini Kit (Qiagen; Hilden, Germany) according to the manufacturer’s protocol. For RNA-Seq, 1.5 ug of total RNA per sample was submitted to the Next Generation Sequencing Core at MD Anderson Cancer Center for RNA-seq analysis. The raw sample reads were compared with the human reference genome build HG19 and analyzed using the HTSeq software. Finally, the count data from HTSeq was applied to DESeq to test for differential gene expression. The upregulated genes were characterized for their biological functions using the Database for Annotation, Visualization, and Integrated Discovery (DAVID).

### 2.4. Cell assays

MDA-GSCs were plated at 50,000 cells/well into 12-well plates at and allowed to grow for 5 days until neurospheres were formed. The cells were then treated with either TCP, GSK-LSD1, or RN-1 at various concentrations for 5 days. Next, the cells were collected and neurospheres were dissociated with Accutase (BioLegend; San Diego, CA). Cell viability was determined using a trypan dye exclusion assay and read with the Vi-Cell XR Cell Viability Analyzer (Beckman Coulter; Brea, CA). NHAs were plated at 50,000 cells/well into 12-well plates and allowed to adhere overnight. The following day, NHAs were treated with the LSD1 inhibitors. After 5 days, the cells were detached using TrypLE and assessed with the trypan blue dye exclusion assay.

The alamarBlue cell viability (Bio-Rad; Hercules, CA) was performed on the MDA-GSCs according to the manufacturer’s protocol. The cells were plated at 5,000 cell/well into a 96-well plate and allowed to grow for 5 days. After 5 days, the alamarBlue reagent was added and fluorescence was measured with the Synergy 2 (BioTek Instruments; Winooski, VT) plate reader according to the manufacturer’s protocol.

Colony formation assay was performed on MDA-GSC 311, MDA-GSC 20, MDA-GSC 23, and MDA-GSC 8–11. Cells were plated in triplicate into clear, flat-bottom 96-well plates at 3 cells/well into 100 μL of media. MDA-GSCs were treated with 100 μL of GSK-LSD1 and allowed to grow for 3 weeks at 37°C before scoring. The final concentration of GSK-LSD1 in each well was determined using the IC_50_ in each cell line (MDA-GSC 311 at 200 μM, MDA-GSC 20 at 300 μM, MDA-GSC 23 at 200 μM, MDA-GSC 8–11 at 400 μM). Clonogenicity was scored using the Cell3iMager (Screen Life Science; Elk Grove Village, IL) to count the number of wells with colony formation as positive and wells without colony formation as negative.

### 2.5. Western blot analysis

Total protein from each MDA-GSC line was extracted using RIPA buffer and quantified using the Bradford assay. Equal amounts of protein, 50 μg, were loaded onto a 10% polyacrylamide gel. PVDF membranes were blocked with 1x TBST with 5% milk and probed with anti-LSD1 (Abcam #17721) and anti-actin (Millipore Sigma #A2066). Chemiluminescent images were detected with electrochemiluminescence (ECL) reagents, imaged with ChemiDoc, and quantified with Image Lab (Bio-Rad; Hercules, CA).

### 2.6. *In vivo* experiments

All animal experiments were approved by the University of Texas MD Anderson Cancer Center Institutional Animal Care and Use Committee (IACUC) under protocol 638 (P.I. Chandra). An equal number of male and female athymic nude mice aged 4–6 weeks old were bolted with guide screws by MD Anderson’s Brain Tumor Center Animal Core as previously described (16). Ketamine/xylazine was administered for anesthesia and buprenorphine or buprenorphine sustained-release (SR) was administered for analgesia during and after the surgery. MDA-GSC20 luciferase labelled cells were resuspended in PBS and 500,000 cells were intracranially implanted into each mouse *via* guide screw. To monitor tumor engraftment and tumor progression throughout treatment, each mouse was imaged weekly using an IVIS 200 imager (Perkin Elmer; Waltham, MA). Mice were anesthetized *via* inhalation of isoflurane and injected with 200 μL of d-Luciferin (GoldBio; St Louis, MO) subcutaneously. Bioluminescence signal in the mice was read and reported as photons/s.

Once confirmation of intracranial tumor engraftment at 7 days post-engraftment, the mice were equally randomized into groups to receive either GSK-LSD1 or the vehicle control. The mice received GSK-LSD1 1 mg/kg diluted in DMSO and PBS (5 and 95%, respectively) *via* intraperitoneal injection (200 μL/mouse) while the control mice received the untreated vehicle in parallel. In accordance with the approved IACUC protocol (No: 638-RN03) at MD Anderson Cancer Center, treatment continued until the mice succumbed to tumor burden and were found in a moribund condition as determined by neurological impairment (hydrocephalus, seizures, inactivity, and/or ataxia) or morbidity (hunched posture or weight loss). At this time mice were euthanized by both carbon dioxide exposure and cervical dislocation. Flow cytometric characterization of immune cell subsets was carried out in blood samples obtained from mice (four control mice and six GSK-LSD1 mice) in each group at day 50 *via* cardiac puncture and the sample was processed using Ficoll (GE Healthcare; Chicago, IL) centrifugation to collect the peripheral blood mononuclear cells (PBMCs). Next, PBMCs were stained with the Ghost Dye Violet (Tonbo; San Diego, CA) and then incubated with antibodies at 4°C for 25 min before analysis. The antibodies anti-NKG2D APC (Biolegend #320807), CD16-PE-Cy7 (ThermoFisher #25–0168-42), CD56-PE (BD Biosciences #345812), anti-CD3-FITC (Biolegend; #100305), CD4-PerCP (ThermoFisher #MHCD0431), anti-CD8-ACP-Cy7 (Biolegend #344713), and anti-SLAMF7-PE (eBioscience #12–2,229-42) were used in concentrations recommended per manufacturer’s instruction. Samples were washed and resuspended in FACS buffer for analysis on a Fortessa flow cytometer (BD Biosciences; Haryana, India). Compensation was achieved with UltraComp bead (ThermoFisher; Waltman, MA) and the BD FACSDiva software.

### 2.7. qPCR

Total RNA was extracted using RNeasy Plus Mini Kit (Qiagen; Hilden, Germany) following the manufacturer’s protocol. cDNA was synthesized with the iScript cDNA synthesis kit (Bio-Rad; Hercules, CA), qPCR was performed using Forget-Me-Not EvaGreen qPCR kit (Biotium; Fremont, CA), and samples were analyzed with the LightCycler96 (Roche; Basel, Switzerland). Cq values were normalized to PPIA and reported as relative fold change from untreated control. Primers to determine mRNA expression were used as shown in Table 4.

**Table 4:** Primer sequences for qPCR experiments.

Primer	Forward	Reverse
FTH1	AGGTGCGCCAGAACTACCAC	TCAAAGCCACATCATCGCGG
RAB39B	CTCGCGCCTACTACAGGAAC	CACACTTGTGACCCACCAGA
HKDC1	GGTGGATGAGGGGTCCTTGA	CCGGAGACGCTCTGAAATCT
FAM213A	GGGCCGGGCGGTTTG	AGGAAAGACATTTCTCAAGTGCCTC
PPIA	CCCACCGTGTTCTTCGACATT	GGACCCGTATGCTTTAGGATGA

### 2.8. TCGA analysis

The glioblastoma dataset “Glioblastoma multiforme (TCGA, PanCancer Atlas)” from the cBioPortal for cancer genomics (17, 18) was probed for the expression of five genes correlated to resistance to LSD1 inhibitors. Supervised clustering of the samples with mRNA expression data was performed for LSD1 and the five genes.

### 2.9. Data analysis

All experiments were conducted in triplicate as biological replicates and within each experiment as technical replicates. Values are presented as mean ± standard deviation (SD) unless otherwise stated. The graphs were generated using GraphPad Prism 9. Unpaired student’s t-tests were used to compare the means of two samples. The one-way ANOVA with Tukey’s multiple comparisons was used to compare sample means with three or more groups. Two-way ANOVA or a mixed-effects analysis with the Šídák multiple comparisons test was used to compare samples with two independent variables. A value of *p* < 0.05 was used to determine significance between samples.

## 3. Results

### 3.1. Transcriptomic evaluation of LSD1 knockout versus pharmacological inhibition in glioblastoma

**Figure 1:**
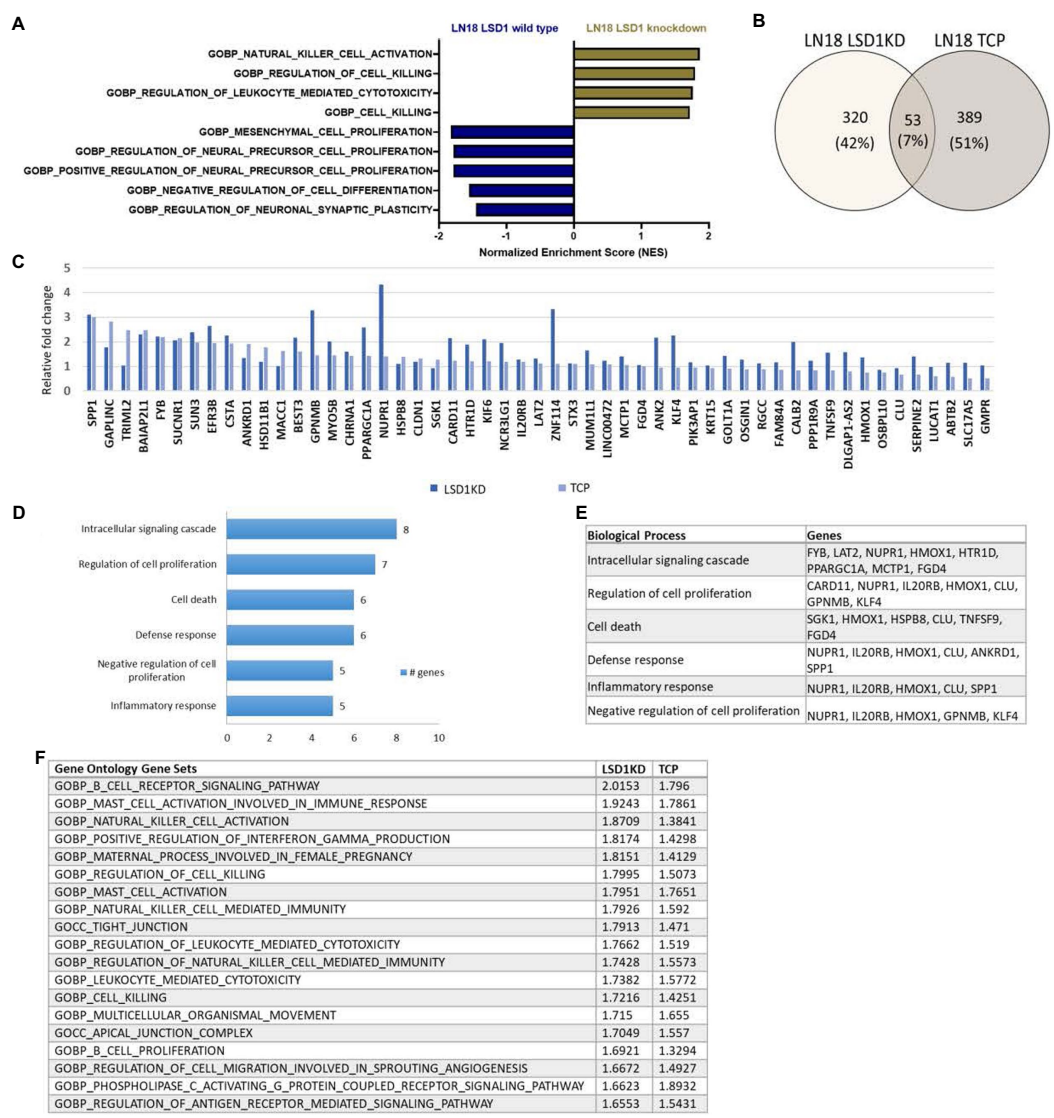
Inhibition of LSD1 and the associated processes. **(A)** GSEA of LN18 glioblastoma cells was used to compare knockdown of LSD1 with wild type LSD1. False discovery rate of <25% was used to determine significance. **(B)** Venn diagram showing overlapping changes in upregulated gene expression upon TCP treatment and LSD1KD in LN18 cells. **(C)** List of common genes upregulated from TCP treatment and LSD1KD in LN18 cells. **(D)** Ontology analysis of the upregulated genes from either TCP treatment or LSD1KD showing relevant categories from the biological processes classifications. **(E)** List of genes from each biological process classification of the ontology analysis. **(F)** GSEA showing the list of overlapping gene sets from the gene ontology gene set collection and the respective normalized enrichment scores for each analysis. False discovery rate of <25% was used to determine significance.

To further understand the biological implications of LSD1 inhibition in GBM, we evaluated the transcriptional effect of LSD1 knockdown in a human GBM cell line, LN18. Differential gene expression of LSD1 knockdown was compared with LSD1 wild type using gene set enrichment analysis (GSEA) (19–21). The LSD1 wild type phenotype is enriched with genes corresponding to gene sets for the regulation of neural precursor cell proliferation, mesenchymal cell proliferation, and negative regulation of cell differentiation (Figure 1A). In contrast, the LSD1 knockdown phenotype is enriched with genes that represent gene sets for regulation of cell killing and regulation of leukocyte-mediated cytotoxicity (Figure 1A). Next, we compared the transcriptional profile of LSD1 knockdown with pharmacological inhibition of LSD1 *via* TCP (1 mM) in LN18 cells. Knockdown of LSD1 and inhibition by TCP resulted in the upregulation of hundreds of genes, many of which were unique to LSD1 knockdown or TCP treatment alone. There was a subset of the upregulated genes, including 53 genes, that were similarly upregulated across LSD1 knockdown and TCP treatment (Figures 1B,C). The 53 overlapping genes were then examined in a gene ontology analysis of biological processes using DAVID to classify the processes associated with the commonly upregulated genes. The results from the gene ontology analysis demonstrate that LSD1 inhibition effects several important processes such as regulation of intracellular signaling, regulation of cell proliferation, and cell death (Figures 1D,E). Importantly, these biological implications of LSD1 inhibition are shared across gene knockdown and pharmacological inhibition. The differential gene expression data of LSD1 knockdown and TCP treatment was further analyzed by GSEA. This analysis revealed 19 overlapping gene sets that were commonly enriched for both knockdown and pharmacological inhibition (Figure 1F). These data highlight genes and processes in human GBM regulated by both LSD1 knockdown and by a first-generation LSD1 inhibitor, TCP.

### 3.2. Assessment of efficacy of LSD1 inhibitors in GSC models

**Figure 2:**
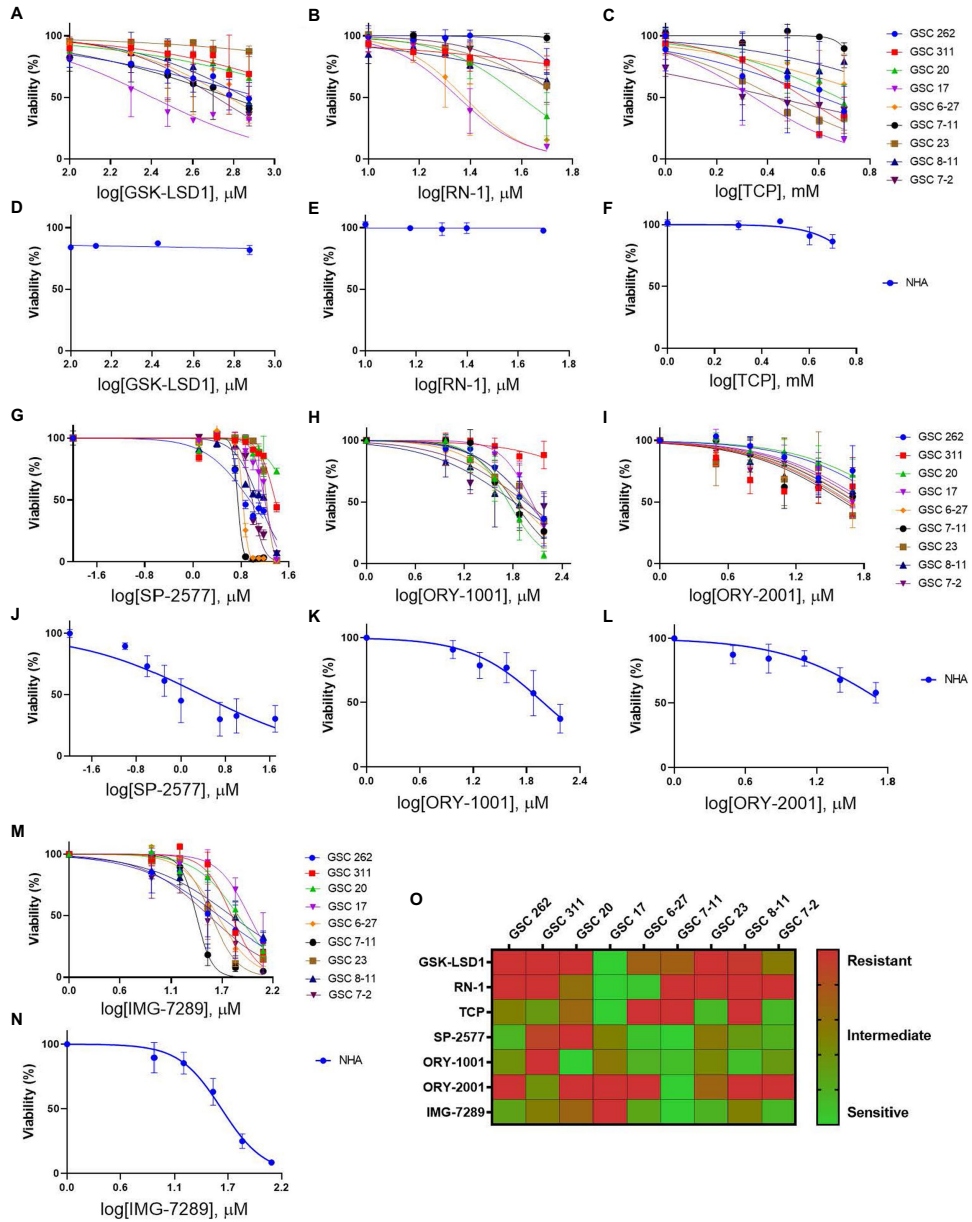
Sensitivity and selectivity of LSD1 inhibitors for GSCs. **(A–C)** Dose response curves for MDA-GSCs and **(D–F)** NHAs treated with GSK-LSD1, RN-1, and TCP. **(G–I)** Dose response curves for MDA-GSCs and **(J–L)** NHAs treated with SP-2577, ORY-1001, ORY-2001. **(M)** Dose response curves for MDA-GSCs and **(N)** NHAs treated with IMG-7289. All experiments were completed in triplicate. **(O)** Heat map showing the treatment response of MDA-GSCs to the corresponding inhibitor.

To evaluate the efficacy of additional pharmacological inhibitors of LSD1 in MDA-GSCs, seven small molecule LSD1 inhibitors were assessed in a panel of nine MDA-GSC lines. This panel of MDA-GSCs represents the diverse characteristics of glioblastoma and includes lines that are radiosensitive, radioresistant, and the three major molecular subtypes (proneural, classical, and mesenchymal). Six irreversible, catalytic LSD1 inhibitors, including TCP, GSK-LSD1, RN-1, ORY-1001, ORY-2001, and IMG-7289, and one scaffolding inhibitor, SP-2577, were evaluated in our MDA-GSC models. GSK-LSD1 affected MDA-GSC viability in many of the MDA-GSC lines at concentrations ranging from 250 to 750 μM (Figure 2A). Likewise, RN-1 was active and reduced the MDA-GSC viability in the range of 20–50 μM (Figure 2B). Effects on cell viability with TCP treatment were not seen until mM concentrations (Figure 2C), yet are clinically relevant (22). The selective effects of LSD1 inhibition for MDA-GSCs was demonstrated by screening the compounds in noncancerous brain cells, including immortalized NHA. Treatment with GSK-LSD1, RN-1, and TCP had minimal effect on the NHA when treated at the maximum concentration tested for each compound (Figures 2D–F). Interestingly, the scaffolding inhibitor, SP-2577, demonstrated the most potent effects on cell viability in several MDA-GSC lines, with strong effects at less than 10 μM (Figure 2G). We extended our studies to include several LSD1 inhibitors which have been tested in clinical trials for liquid and non-neural solid tumors (23–25). Treatment with ORY-1001 resulted in a reduction in cell viability in several MDA-GSC lines at concentrations ranging from 50 to 90 μM (Figure 2H). In contrast to ORY-1001, ORY-2001 was not as potent in our panel of MDA-GSCs since the majority did not reach an IC50 at the maximum dose of 50 μM (Figure 2I). While SP-2577 had potent effects in the MDA-GSC lines, it also demonstrated strong activity in the noncancerous cells, NHA (Figure 2J), and this compound has not been found to cross the blood brain barrier (personal communications, Salarius Pharmaceuticals). Effects on NHA viability were assessed for ORY-1001 and ORY-2001 using dose ranges applied in the MDA-GSC models (Figures 2K,L). Lastly, treatment with IMG-7289 caused a decrease in MDA-GSC viability in concentrations <50 μM (Figure 2M) and was its effects were assessed on NHA as well (Figure 2N). The half maximal inhibition concentration (IC_50_) of the seven LSD1 inhibitors in individual MDA-GSC lines compared with the noncancerous brain cell lines are shown cumulatively and demonstrate the sensitivity and selectivity of LSD1 inhibitors for several MDA-GSC lines (Table 5). The response to LSD1 inhibitors, based on the IC_50_, indicates that there are differential cytotoxic effects of LSD1 inhibition. These effects are dependent on the MDA-GSC line and given LSD1 inhibitor as depicted by the heatmap (Figure 2O). The MDA-GSC lines were compared to each other to define their relative sensitivity or resistance to individual LSD1 inhibitors. For example, there is only one MDA-GSC line, MDA-GSC17, that is sensitive to all four LSD1 inhibitors. Additionally, each LSD1 inhibitor had some MDA-GSCs that were sensitive to the treatment and others that were resistant and were largely non-overlapping. These data demonstrate the unique biological effects of each LSD1 inhibitor in various MDA-GSC models.

**Table 5:** Comparison of LSD1 inhibitor effect on cell viability.

	GSK-LSD1 (μM)	RN-1 (μM)	TCP (mM)	SP-2577 (μM)	ORY-1001 (μM)	ORY-2001 (μM)	IMG-7289 (μM)
MDA-GSC 262	>750	>50	4.1	8.8	92.7	>50	41.2
MDA-GSC 311	>750	>50	3.5	23.6	>150	45.6	>125
MDA-GSC 20	>750	39.8	4.7	>25	54.8	>50	66.9
MDA-GSC 17	253.4	22.5	2.2	15.7	106.9	>50	87.4
MDA-GSC 6–27	598.5	23.8	>5	6.8	72.8	>50	39.9
MDA-GSC 7–11	606.3	>50	>5	5.6	64.7	42.1	23.6
MDA-GSC 23	>750	>50	2.7	16.8	97.8	48.1	34.2
MDA-GSC 8–11	726	>50	>5	12.4	64.5	>50	56.1
MDA-GSC 7–2	518.5	>50	2.7	9.7	87.7	>50	32.1
NHA	>750	>50	>5	2.3	96.3	>50	38.2

The IC50 values represent the mean of three independent cell viability experiments from the dose response curves shown in Figure 2.

**Figure 3:**
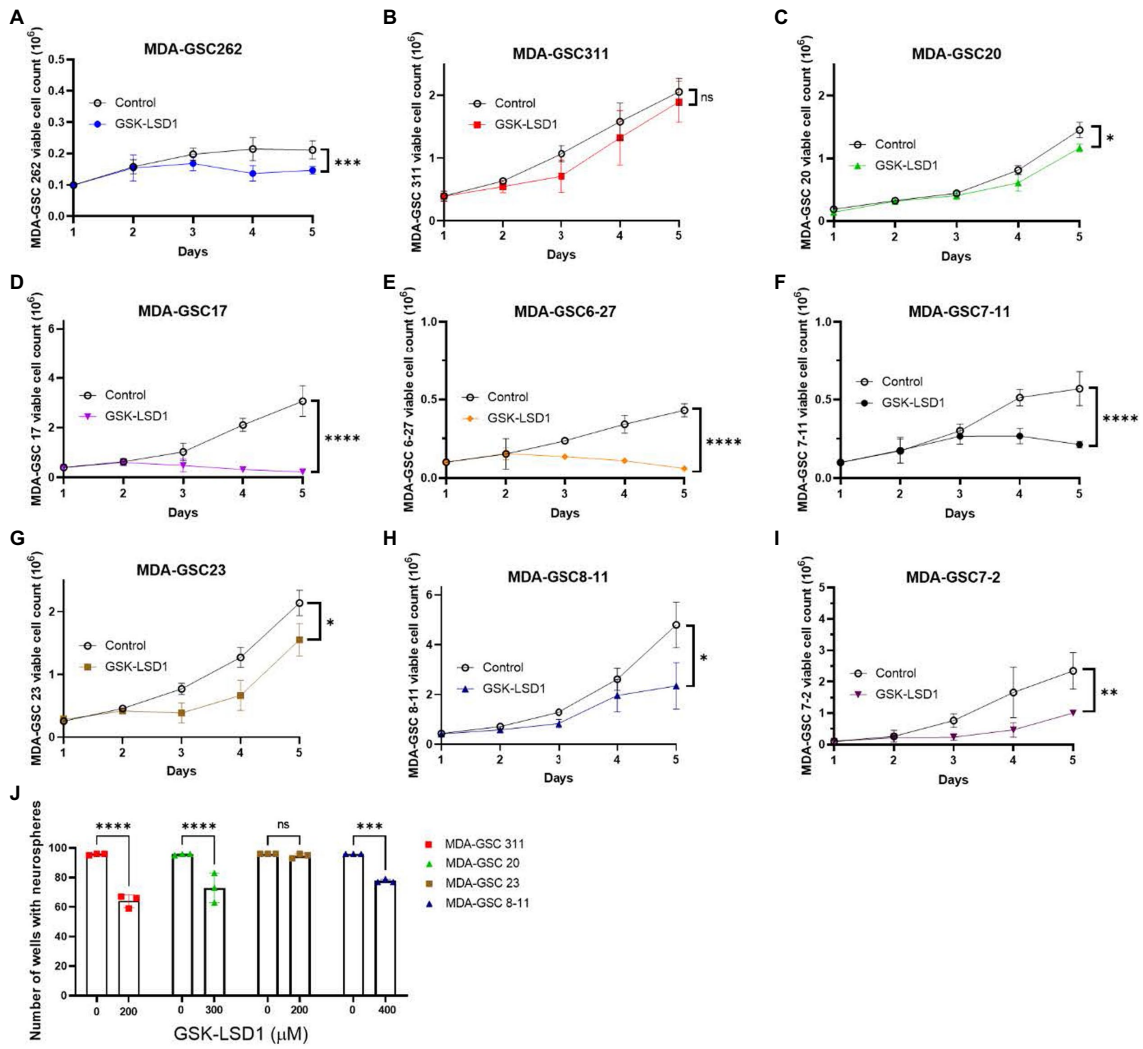
Effects of GSK-LSD1 on GSCs cell proliferation and neurosphere formation. **(A–I)** Cell proliferation of MDA-GSCs was tracked over 5 days after treatment with GSK-LSD1 at 400 μM. Day five data was analyzed with a student’s *t*-test. ns = *p* > 0.05, *p ≤ 0.05, **p ≤ 0.01, ***p ≤ 0.001, ****p ≤ 0.0001. **(J)** Bar graphs showing the number of neurospheres formed by MDA-GSCs after treatment with GSK-LSD1. Grouped data was analyzed using a two-way ANOVA with the Šídák multiple comparisons test. ns = *p* > 0.05, ***p ≤ 0.001, ****p ≤ 0.0001.

Next, the LSD1 inhibitor, GSK-LSD1, was selected for further evaluation of effects on proliferation due to its wide therapeutic window that has been previously demonstrated in multiple CNS tumors and confirmed in our MDA-GSC lines (26, 27). First, the effect of GSK-LSD1 on MDA-GSC proliferation was assessed in all nine of the MDA-GSC lines (Figures 3A–I). Consistent anti-proliferative effects after 5 days were observed with all the MDA-GSC lines, apart from MDA-GSC311. Similar to the dose response curves, the MDA-GSC17, 6–27, and 7–11 show large differences from treatment of GSK-LSD1.

Four GSK-LSD1-resistant MDA-GSC lines, defined by the IC_50_, were evaluated to assess the effect of GSK-LSD1 on MDA-GSC colony formation. In the presence of GSK-LSD1, three of the four cell lines, MDA-GSC311, MDA-GSC20, MDA-GSC23, and MDA-GSC8-11, have a reduced capacity to form neurospheres from single cells (Figure 3J). Therefore, while GSK-LSD1 may not have profound effects on cell viability in these MDA-GSC lines, it can still limit MDA-GSC survival and disrupt their clonogenic potential. These effects are particularly important for the MDA-GSC20 line, as it models radioresistance and it is the mesenchymal molecular subtype that is associated with the worst survival outcomes (28, 29). Overall, these data highlight the ability of the MDA-GSC models to capture elements of GBM tumor heterogeneity, caveats of which include distinct growth rates and need for prolonged exposure to GSK-LSD1.

**Figure 4:**
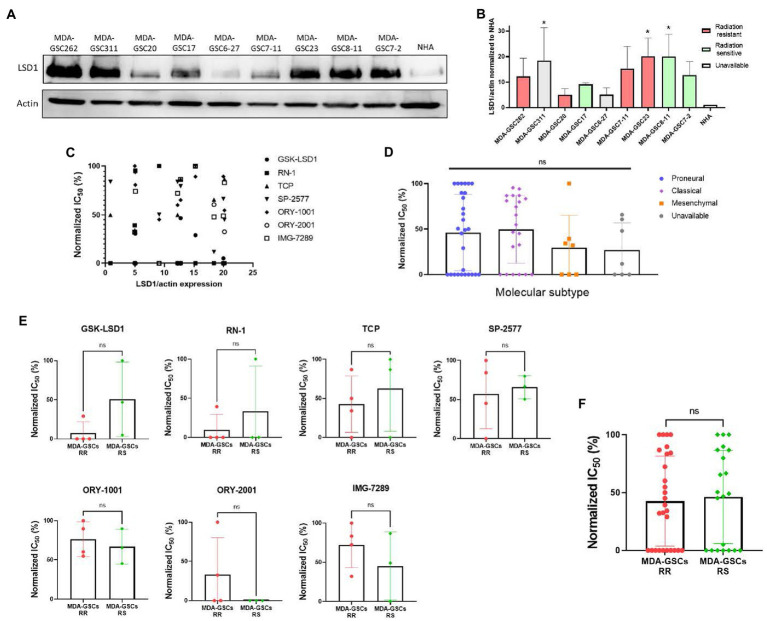
Sensitivity to LSD1 inhibitors is not predicted by LSD1 expression, GSC radiation sensitivity, or TCGA subtype. **(A)** Representative Western blot showing the basal expression level of LSD1 protein in MDA-GSC lines from three independent experiments. **(B)** Quantification of LSD1 expression normalized to actin and compared to the NHA. Statistical analysis performed by one-way ANOVA with Dunnett’s multiple comparsons test to compare the mean LSD1 expression of the MDA-GSC lines to NHA. *p ≤ 0.05. **(C)** Scatter plot assessing the correlation between LSD1 protein expression and treatment response to the LSD1 inhibitors measures with a Pearson correlation coefficient. **(D)** Summary graph comparing the treatment response to LSD1 inhibitors based on TCGA subtype of each MDA-GSC. Statistical analysis performed by one-way ANOVA. **(E)** Comparison of MDA-GSC radiation sensitivity status based on the treatment response to individual LSD1 inhibitors. Statistical analyses performed by unpaired *t*-test. **(F)** Summary graph comparing the treatment response to LSD1 inhibitors based on MDA-GSC radiation sensitivity. Statistical analysis performed by by unpaired *t*-test. ns = *p* > 0.05.

To investigate tumor characteristics that may predict a response to LSD1 inhibition, we compared the response to LSD1 inhibitors with LSD1 expression, MDA-GSC sensitivity to radiation, and the molecular subtype. LSD1 protein expression from patient-derived MDA-GSC lines was measured by western blotting and quantified by densitometry (Figures 4A,B). Protein expression was then compared with the MDA-GSC sensitivity to LSD1 inhibitors to examine if LSD1 expression correlated with response. From this analysis, there is no correlation between LSD1 protein expression and the IC_50_ of individual MDA-GSC lines, Pearson *r* = −0.3913 (Figure 4C). Furthermore, we considered the sensitivity to LSD1 inhibitors in relation to the sensitivity to radiation therapy or the molecular subtype. Our results show that sensitivity to LSD1 inhibition is not predicted by the molecular subtype classifications of glioblastoma (Figure 4D). Similarly, there is no profound difference in sensitivity, based on radiosensitivity, to any LSD1 inhibitor alone (Figure 4E), nor to LSD1 inhibition as an entire group (Figure 4F). Overall, single agent LSD1 inhibition shows promise in many MDA-GSC lines, but the sensitivity is not dependent on established tumor characteristics such as radiosensitivity or categorization into GBM subtypes.

### 3.3. *In vivo* inhibition of LSD1 promotes reduction in tumor burden

**Figure 5:**
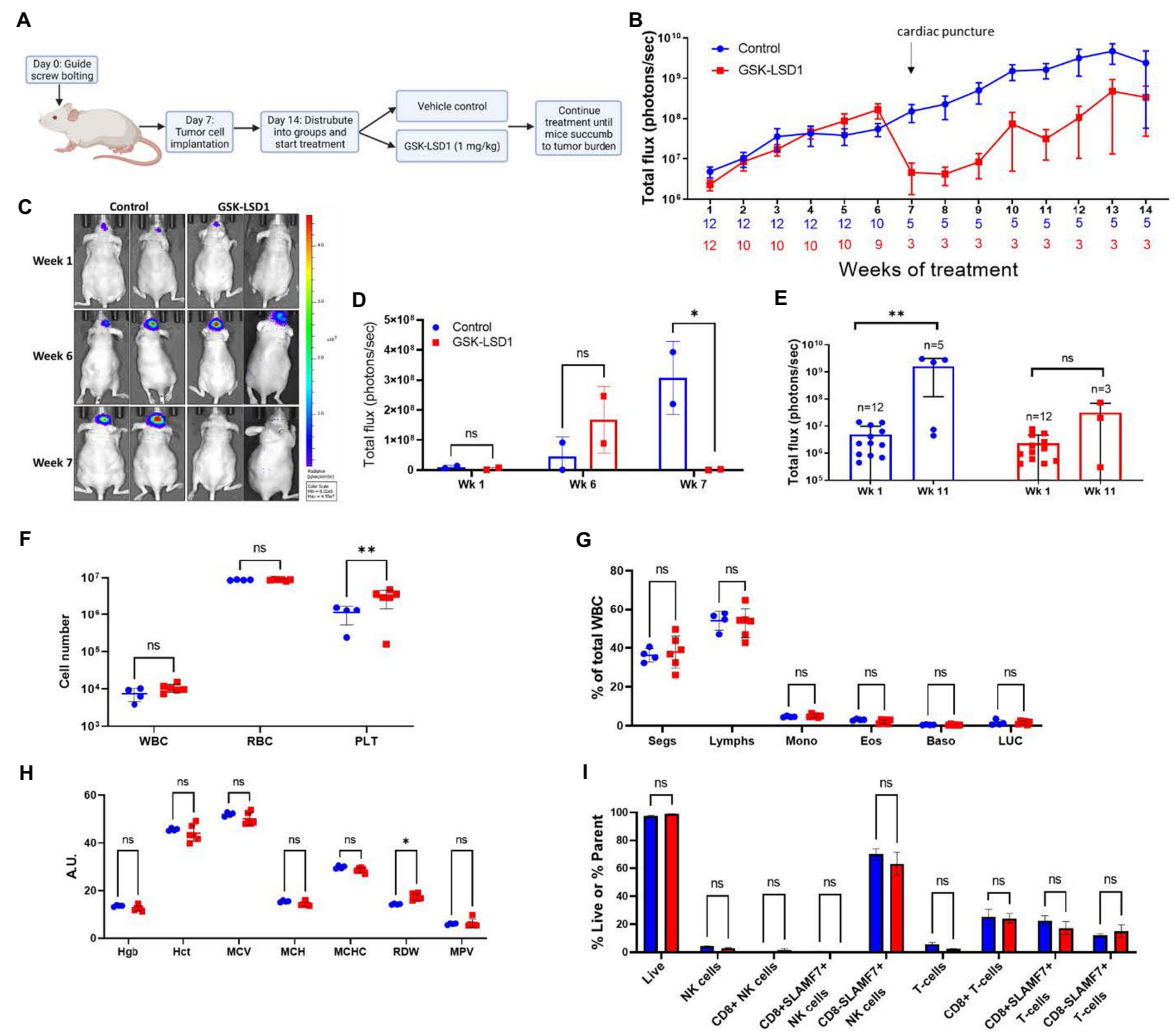
Activity of LSD1 inhibition in a radioresistant GSC model. **(A)** Timeline of *in vivo* experiments with orthotopic xenografts of MDA-GSC 20. **(B)** Serial imaging was performed weekly throughout the study to measure bioluminescene as an indicator of tumor progression (*n* = 12 mice per group). Bioluminescence is quantified as total flux. Error bars indicate ± standard error of the mean (SEM). **(C,D)** Mice at week 1, week 6, and week 7 post-tumor engraftment to compare the effects of GSK-LSD1 with the vehicle. The luminescence signal is quantified as total flux and displayed as mean ± SD. Data was analyzed with a two-way ANOVA with the Šídák multiple comparisons test. **(E)** Comparison of tumor progression at week 1, pre-treatment, and week 11, post-treatment. Individual values are plotted and and statistical analysis performed by mixed-effects analysis with the Šídák multiple comparisons test. Data from tumor progression is an accumulation of data from two independent experiments. **(F)** Complete blood count from mice 50 days post-treatment with **(G)** white blood cell differential and **(H)** red blood cell measurements (*n* = 4 mice per control and *n* = 6 mice per GSK-LSD1). **(I)** Analysis of peripheral blood natural killer cell and T-cell subsets from mice 50 days post-treatment. Data collected for the blood samples were analyzed with a two-way ANOVA with the Šídák multiple comparisons test. Error bars indicate ± SEM. ns = *p* > 0.05, *p ≤ 0.05, **p ≤ 0.01.

An intracranial orthotopic xenograft model of MDA-GSC20 cells was used to evaluate efficacy of GSK-LSD1 based on its effects on MDA-GSC clonogenicity and cell proliferation. One week after tumor implantation *via* guide screw, mice had confirmed tumor engraftment as measured by bioluminescence and were stratified into two groups to receive either vehicle control or GSK-LSD1 (Figure 5A). Following the start of treatment, mice were imaged weekly to monitor tumor progression throughout the duration of treatment (Figure 5B). Tumor burden steadily increased in both groups through week six, however; at week seven, a strong regression in tumor burden was observed in the group of mice receiving GSK-LSD1, but not the control. Two mice in the GSK-LSD1 group showed a decrease in tumor burden when compared with the control mice (Figures 5C,D). The effect of the GSK-LSD1 on tumor burden persisted into week 11 (Figure 5E). At week 11 it is apparent that the mice receiving GSK-LSD1 treatment have a lower rate of tumor progression compared with the control mice. However, beyond week 11, tumor growth resumes and continued dosing with GSK-LSD1 is ineffective to improve overall survival.

Given the effect on tumor regression required almost 2 months of treatment, we sought to determine if any hematological toxicities were present from the prolonged exposure to LSD1 inhibition. At day 50, a subset of mice from each group were sacrificed and blood was collected for evaluation with a Complete Blood Count (CBC) to compare different populations of blood cells. Importantly, there is no evidence of leukopenia or erythropenia in the GSK-LSD1 treated mice compared to the untreated mice (Figure 5F). However, the platelet count in the GSK-LSD1 receiving mice is higher compared to the control and may be secondary to the underlying tumor burden (Figure 5F). The WBC differential further confirmed the absence of hematological toxicity with no difference between the GSK-LSD1 and the control group in any of the individual cell types (Figure 5G). Evaluation of the RBC indices largely demonstrate no effect from GSK-LSD1 except for red cell distribution width (RDW) (Figure 5H). In parallel, the peripheral blood mononuclear cells (PBMCs) were also isolated and probed to assess the effect of GSK-LSD1. Prior studies of LSD1 inhibition *via* pharmacological inhibitors prompted the assessment of GSK-LSD1 effect on different subpopulations of natural killer (NK) cells and T-cells (26, 30). Importantly, our results indicate that the LSD1 catalytic inhibitor, GSK-LSD1, does not have cytotoxic effects on NK or T-cells (Figure 5I). Overall, the assessment of hematological parameters demonstrates no overt effects from GSK-LSD1 under our study conditions.

### 3.4. Resistance to LSD1 inhibition

**Figure 6:**
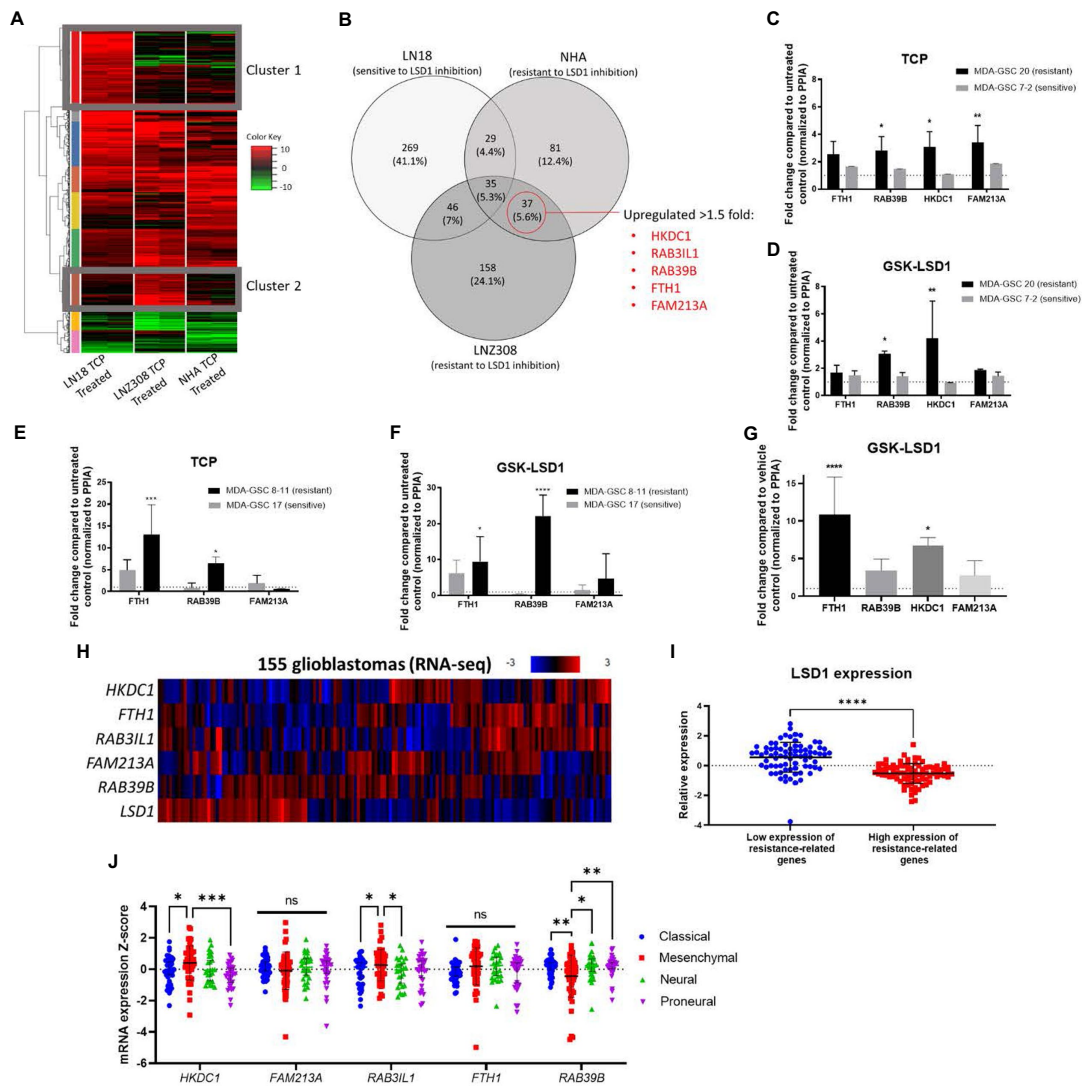
Identification of a gene set that correlates with resistance to LSD1 inhibition. **(A)** Heat map of RNA-Seq analysis from three cell lines treated with TCP. Cluster 1 represents genes that are upregulated in a LSD1 inhibitor sensitive line, LN18, but not LNZ308 or NHA. Cluster 2 genes are upregulated in LSD1 inhibitor resistant lines, LNZ308 and NHA, but not LN18. **(B)** Venn diagram emphasizing the similarly upregulated genes from cell lines resistant to TCP. Five genes were chosen for their potential as predictors of LSD1 inhibitor treatment outcome. **(C)** Bar graphs of gene expression upon TCP treatment and **(D)** GSK-LSD1 treatment in MDA-GSCs. **(E)** Bar graphs of gene expression upon TCP treatment and **(F)** GSK-LSD1 treatment in an additional set of MDA-GSCs resistance/sensitive to LSD1 inhibition. **(G)** Bar graph of gene expression from the brain tissue of mouse treated with GSK-LSD1 compared to representative control mouse. Gene expression data was analyzed with a two-way ANOVA with the Šídák multiple comparisons test. **(H)** Heat map of RNA-seq data from glioblastoma patients probed for the select resistance-related genes. **(I)** LSD1 expression in patients with low and high expression of the genes correlating with resistance. Statistical analyses performed by students *t*-test. *p ≤ 0.05, **p ≤ 0.01, ****p ≤ 0.0001. **(J)** Interleaved scatter plot of resistance related genes’ expression compared across GBM subtypes. Statistical analysis performed by two-way ANOVA with Fisher’s LSD test. ns = not significant, *p ≤ 0.05, **p ≤ 0.01, ***p ≤ 0.001.

Given the transient efficacy of LSD1 inhibition *via* GSK-LSD1 in the MDA-GSC20 xenograft model, we sought to determine which genes may predict resistance to LSD1 inhibitor treatment. First, we established an *in vitro* model using one cell line that was sensitive to LSD1 inhibition, LN18, and two cell lines resistant to LSD1 inhibition, NHA and LNZ308 (31). Upon TCP (1 mM) treatment, we examined the changes in gene expression in each cell line. TCP was chosen for the initial assessment, as it is the prototypical small molecule LSD1 inhibitor. Using RNA-Seq, we defined two gene clusters of particular interest (Figure 6A). Cluster 1 represents genes that are only upregulated in the LSD1 inhibitor sensitive line. In contrast, Cluster 2 genes are only upregulated in LSD1 inhibitor resistant lines. The genes were sorted into groups based on overlapping expression profiles (Figure 6B). Five genes from Cluster 2 were chosen as potential biomarkers that may predict response to LSD1 inhibition. The rationale for the selection of the genes, *HKDC1*, *RAB3IL1*, *RAB39B*, *FTH1*, and *FAM213A*, is their potential pro-tumorigenic roles in other cancer types. For example, HKDC1 has been implicated in tumorigenesis of lung adenocarcinoma through modulation of AMPK/mTOR signaling (32). *RAB3IL1* encodes a guanine exchange factor that binds to the RAS-related protein, RAB3A. RAB39B, a Ras-analog in brain (RAB) small GTPase, is a member of the RAS oncogene family and is involved in vesicular trafficking (33). High expression of ferritin heavy chain 1 (FTH1) has been associated with high-grade gliomas and poor prognosis in glioblastoma (34, 35). Lastly, the redox regulatory protein, FAM213A, has shown to activate antioxidant proteins and elevated expression is associated with worse outcomes for AML patients (36).

Next, to evaluate the proposed genes that correlate with resistance to LSD1 inhibition, the expression these genes were measured in MDA-GSCs treated with TCP and GSK-LSD1. For TCP, the MDA-GSC20 and MDA-GSC7-2 lines were selected as representatives for TCP resistance and sensitivity, respectively. The gene expression of the select genes were consistently increased in the MDA-GSC20 line, whereas there were only minimal effects of TCP on these same genes seen in the MDA-GSC7-2 line (Figure 6C). This trend was also found after GSK-LSD1 treatment on MDA-GSC20, resistant, and MDA-GSC7-2, sensitive. After GSK-LSD1 treatment, the MDA-GSC20 cells showed an increase in all four genes and the MDA-GSC7-2 again had inappreciable effects (Figure 6D). Only four of the five resistance genes were measured in these GSC lines because there was too little expression of *RAB3IL1* to detect. These results were repeated in an additional pair of sensitive and resistance MDA-GSC lines, MDA-GSC17 and 8–11, respectively. In response to TCP, the resistant MDA-GSC8-11 line had an increase in two of the resistance related genes, while the sensitive MDA-GSC17 did not significantly increase (Figure 6E). Likewise, treatment with GSK-LSD1 showed an increase in the same two gene in the MDA-GSC8-11 line, but not the MDA-GSC17 line (Figure 6F). There was low expression of *HKDC1* and *RAB3IL1* in the MDA-GSC8-11 and 17 lines and therefore were not reported. Overall, when considering the MDA-GSC transcriptional response to LSD1 inhibition, the identified gene set appears conserved for TCP and GSK-LSD1.

We then measured the gene expression of the candidate gene set in brain tissue of a mouse treated with GSK-LSD1 compared to a control mouse after they succumbed to tumor burden. In these mice, three of the four genes, except for *FAM213A*, were upregulated in the GSK-LSD1 treatment mouse (Figure 6G). Finally, we assessed the presence of this gene set in patient data acquired from TCGA (17, 18). In a dataset of glioblastoma patient samples, LSD1 expression has an inverse relationship with the genes that correlate with resistance (Figures 6H,I). We then assessed the correlation between resistance related gene expression and GBM subtypes using TCGA (Figure 6J). From this analysis, we find that the mesenchymal subtype has an increased expression of *HKDC1* and *RAB3IL1* compared to classical and proneural. Interestingly, the mesenchymal subtype also has reduced expression of *RAB39B* compared to proneural, neural, and classical. Overall, our established gene set may predict resistance to LSD1 inhibition and is concordant across *in vitro* experiments, *in vivo* studies, and is expressed in a LSD1 dependent manner in GBM patients.

## 4. Discussion

This study builds upon previous literature surrounding the evaluation of LSD1 inhibitors in various malignancies, including glioblastoma, and expands our past research on LSD1 inhibitors into patient-derived models of glioblastoma (21, 26, 31, 37). We find unique, non-overlapping patterns of *in vitro* efficacy in a diverse panel of patient-derived GBM cell lines representing various ranges of radiosensitivity and genomic and molecular subcategories. Our *in vivo* study demonstrated the delayed activity of GSK-LSD1 after 6 weeks of tumor growth. At week seven we observed a strong regression of tumor burden in the radioresistant MDA-GSC20 orthotopic xenograft. The regression was eventually followed by tumor regrowth which tracked with the expression of genes that are correlated to resistance from our transcriptomic studies. A recently published paper using separate GSC lines and a single LSD1 inhibitor DDP 38003, corroborates these findings and shows tumor regrowth with LSD1 knockdown, supporting a need to understand this process (37). Recently, a direct comparison of pharmacological inhibitors of LSD1 was described in AML and small cell lung cancer models (38), however, our study is the first to extend this comparison into clinically relevant patient-derived MDA-GSC lines. Using the MDA-GSC20 line, a radioresistant GBM model, in an orthotopic xenograft mouse model, a strong effect was observed after 7 weeks of treatment with GSK-LSD1. This delayed time to effect is consistent with other epigenetic therapies such as azacitidine and decitabine in myelodysplastic syndrome and AML (39, 40). Future studies of LSD1 inhibitors should include immunocompetent mouse models for evaluation of efficacy. This type of model may be more permissive for the LSD1 inhibitors to activate immune pathways, which could enhance their *in vivo* effects (as suggested by our prior work and that of others) and mimic a clinical scenario.

Pharmacological inhibitors of LSD1, including TCP, have additional protein targets which can result in varied effects beyond LSD1 inhibition. We observe this effect in the comparison of LSD1 knockdown with TCP treatment in our human GBM line. Here, TCP inhibits monoamine oxidase, in addition to LSD1, and has a relatively low specificity for LSD1 which resulted in a broader impact on the regulation of gene expression. Therefore, it is important to consider the compound-specific effects which gives each LSD1 inhibitor a unique pharmacological profile with varying degrees of efficacy.

One limitation of our study is the use of immortalized NHA as a normal brain counterpart to assess the selective effects of pharmacological LSD1 inhibitors. Human trials of ORY-1001 and IMG-7289 have progressed through extensive safety studies and have not revealed any neurotoxic effects (24, 41). Therefore, it is important to use multiple methods of *in vitro* evaluation when comparing efficacy and to use preclinical *in vivo* experiments to find the maximum tolerated dose and to assess the sensitivity of pharmacological inhibitors. Another limitation of the *in vivo* study is the use of immunocompromised mouse models. Athymic nude mice have a reduced number of T cells, therefore this is not an ideal mouse model for evaluating the effects of GSK-LSD1 on T cells. However, we have previously published the effect of GSK-LSD1 directly on human T cells and found that is T cells are fairly resistant and their cell growth is minimally impacted (26). Future studies will expand the use of LSD1 inhibitors in an immune competent mouse model to validate our hematological findings.

Previous studies have demonstrated that the cell-of origin, either hematopoietic stem cells or myeloid progenitors, influences sensitivity to LSD1 inhibition in leukemia (42). LSD1 inhibitors inhibit the enzymatic activity of LSD1 rather than LSD1 protein expression. Our data indicate that LSD1 protein expression was not a predictor of sensitivity to inhibitors, however we cannot rule out the possibility that post-translational modification or subcellular localization of LSD1was different across the MDA-GSC lines and could influence inhibitor sensitivity. In GBM we modeled tumor regrowth and identified five genes that correlate with resistance to LSD1 inhibition *via* pharmacological inhibition across our GBM models. Importantly, these genes were expressed in our treatment resistant GBM line, GSC lines, and in the brain tissue of mice after tumor regrowth. While we were able to confirm the upregulation of these genes in our resistant GBM models, further investigation with gene knockdown or CRISPR-Cas9 (Clustered Regularly Interspaced Short Palindromic Repeats) is needed to validate each of these five genes as a determinant for treatment resistance in GBM. Studies are currently ongoing to evaluate the effect of each gene alone and their dependencies on one another. Additionally, the identification of these genes and their associated pathways may represent a compensatory pathway that mediates resistance to LSD1 inhibitor. Therefore, upcoming studies will include LSD1 inhibitors in combination with treatments to overcome one or more of these pathways to enhance LSD1 inhibitor efficacy.

The findings of our study contribute to the literature supporting drug development of pharmacological LSD1 inhibitors in cancer therapy. Several LSD1 inhibitors have progressed into clinical trials mainly for the treatment of hematological cancers. Our evaluation of pharmacological LSD1 inhibition in a diverse panel of GSC models suggests the need for future investigations of brain penetrant LSD1 inhibitors alone, or in combination with other therapeutic approaches to synergize efficacy for GBM.

## Data availability statement

The RNA-seq data presented in the study are deposited in the GEO repository, accession number GSE224082.

## Ethics statement

The animal study was reviewed and approved by University of Texas MD Anderson Cancer Center Institutional Animal Care and Use Committee (IACUC) under protocol 638 (P.I. JC). Human MDA-GSC were derived from patients with GBM and established with informed consent after approval from the University of Texas MD Anderson Institutional Review Board under protocol LAB 04-0001.

## Author contributions

LS, AG, LW, DN, AE, MS, KP, YL, XS, RE, JG, SS, ES, FL, and JC participated in research design. LS, AG, LW, DN, AE, MS, and KP conducted the experiments. RE, JG, SS, ES, and FL generated new cell lines. LS, AG, LW, AE, SS, YL, XS, and JC performed the data analysis. LS, AG, and JC wrote or contributed to writing the manuscript. All authors contributed to the article and approved the submitted version.

## Funding

This work was supported by the National Institutes of Health (R21 NS093387, P50 CA127001, TL1 TR003169, R61/R33NS111058 and P30 CA016672), by the Cancer Prevention and Research Institute of Texas (DP160014) and by the Department of Defense (W81XWH2110728).

## Conflict of interest

The authors declare that the research was conducted in the absence of any commercial or financial relationships that could be construed as a potential conflict of interest.

## Publisher’s note

All claims expressed in this article are solely those of the authors and do not necessarily represent those of their affiliated organizations, or those of the publisher, the editors and the reviewers. Any product that may be evaluated in this article, or claim that may be made by its manufacturer, is not guaranteed or endorsed by the publisher.

## Abbreviations

CBC: complete blood count
CRISPR-Cas9: Clustered Regularly Interspaced Short Palindromic Repeats
DAVID: Database for annotation, visualization, and integrated discovery
EGFRvIII: epidermal growth factor receptor variant III
FAM213A: peroxiredoxin-like 2
FTH1: ferritin heavy chain 1
GBM: glioblastoma
GSC: glioblastoma stem cell
GSEA: gene set enrichment analysis
HKDC1: hexokinase domain containing 1
LSD1: lysine-specific demethylase 1
MGMT: O6-methylguanine-DNA methyltransferase
NHA: normal human astrocytes
NK cells: natural killer cells
PBMCs: peripheral blood mononuclear cells
PPIA: peptidylprolyl isomerase A
RAB: Ras-analog in brain
RDW: red cell distribution width
TCP: tranylcypromine

## References

[1] Wen PY Kesari S. Malignant gliomas in adults. N Engl J Med. (2008) 359:492–507. doi: 10.1056/NEJMra0708126 18669428

[2] Maher EA Furnari FB Bachoo RM Rowitch DH Louis DN Cavenee WK. Malignant glioma: genetics and biology of a grave matter. Genes Dev. (2001) 15:1311–33. doi: 10.1101/gad.891601, PMID: 11390353

[3] Stupp R Mason WP van den Bent MJ Weller M Fisher B Taphoorn MJ. Radiotherapy plus concomitant and adjuvant temozolomide for glioblastoma. N Engl J Med. (2005) 352:987–96. doi: 10.1056/NEJMoa043330 15758009

[4] Lathia JD Mack SC Mulkearns-Hubert EE Valentim CL Rich JN. Cancer stem cells in glioblastoma. Genes Dev. (2015) 29:1203–17. doi: 10.1101/gad.261982.115, PMID: 26109046 PMC4495393

[5] Singh SK Hawkins C Clarke ID Squire JA Bayani J Hide T. Identification of human brain tumour initiating cells. Nature. (2004) 432:396–401. doi: 10.1038/nature03128 15549107

[6] Prager BC Bhargava S Mahadev V Hubert CG Rich JN. Glioblastoma stem cells: driving resilience through chaos. Trends Cancer. (2020) 6:223–35. doi: 10.1016/j.trecan.2020.01.009, PMID: 32101725 PMC8779821

[7] Matarredona ER Pastor AM. Neural stem cells of the subventricular zone as the origin of human glioblastoma stem cells. Therapeutic Implications Front Oncol. (2019) 9:779. doi: 10.3389/fonc.2019.00779, PMID: 31482066 PMC6710355

[8] Suvà ML Rheinbay E Gillespie SM Patel AP Wakimoto H Rabkin SD. Reconstructing and reprogramming the tumor-propagating potential of glioblastoma stem-like cells. Cells. (2014) 157:580–94. doi: 10.1016/j.cell.2014.02.030, PMID: 24726434 PMC4004670

[9] Karakaidos P Verigos J Magklara A. LSD1/KDM1A, a gate-keeper of cancer Stemness and a promising therapeutic target. Cancers (Basel). (2019) 11:1821–1841. doi: 10.3390/cancers11121821, PMID: 31756917 PMC6966601

[10] Zhou A Lin K Zhang S Chen Y Zhang N Xue J. Nuclear GSK3β promotes tumorigenesis by phosphorylating KDM1A and inducing its deubiquitylation by USP22. Nat Cell Biol. (2016) 18:954–66. doi: 10.1038/ncb3396, PMID: 27501329 PMC5026327

[11] Sareddy GR Viswanadhapalli S Surapaneni P Suzuki T Brenner A Vadlamudi RK. Novel KDM1A inhibitors induce differentiation and apoptosis of glioma stem cells via unfolded protein response pathway. Oncogene. (2017) 36:2423–34. doi: 10.1038/onc.2016.395, PMID: 27893719 PMC5526658

[12] Perner F Armstrong SA. Targeting chromatin complexes in myeloid malignancies and beyond: from basic mechanisms to clinical innovation. Cells. (2020) 9:2721. doi: 10.3390/cells9122721, PMID: 33371192 PMC7767226

[13] Kamal MM Sathyan P Singh SK Zinn PO Marisetty AL Liang S. REST regulates oncogenic properties of glioblastoma stem cells. Stem Cells. (2012) 30:405–14. doi: 10.1002/stem.1020, PMID: 22228704 PMC4039365

[14] McCord AM Jamal M Shankavaram UT Shankavarum UT Lang FF Camphausen K. Physiologic oxygen concentration enhances the stem-like properties of CD133+ human glioblastoma cells in vitro. Mol Cancer Res. (2009) 7:489–97. doi: 10.1158/1541-7786.MCR-08-0360, PMID: 19372578 PMC6290460

[15] Jiang H Gomez-Manzano C Aoki H Alonso MM Kondo S McCormick F. Examination of the therapeutic potential of Delta-24-RGD in brain tumor stem cells: role of autophagic cell death. J Natl Cancer Inst. (2007) 99:1410–4. doi: 10.1093/jnci/djm102, PMID: 17848677

[16] Lal S Lacroix M Tofilon P Fuller GN Sawaya R Lang FF. An implantable guide-screw system for brain tumor studies in small animals. J Neurosurg. (2000) 92:326–33. doi: 10.3171/jns.2000.92.2.0326, PMID: 10659021

[17] Gao J Aksoy BA Dogrusoz U Dresdner G Gross B Sumer SO. Integrative analysis of complex cancer genomics and clinical profiles using the cBioPortal. Sci Signal. (2013) 6:pl1. doi: 10.1126/scisignal.2004088 23550210 PMC4160307

[18] Cerami E Gao J Dogrusoz U Gross BE Sumer SO Aksoy BA. The cBio cancer genomics portal: an open platform for exploring multidimensional cancer genomics data. Cancer Discov. (2012) 2:401–4. doi: 10.1158/2159-8290.CD-12-0095, PMID: 22588877 PMC3956037

[19] Mootha VK Lindgren CM Eriksson KF Subramanian A Sihag S Lehar J. PGC-1alpha-responsive genes involved in oxidative phosphorylation are coordinately downregulated in human diabetes. Nat Genet. (2003) 34:267–73. doi: 10.1038/ng1180, PMID: 12808457

[20] Subramanian A Tamayo P Mootha VK Mukherjee S Ebert BL Gillette MA. Gene set enrichment analysis: a knowledge-based approach for interpreting genome-wide expression profiles. Proc Natl Acad Sci U S A. (2005) 102:15545–50. doi: 10.1073/pnas.0506580102, PMID: 16199517 PMC1239896

[21] Singh MM Manton CA Bhat KP Tsai WW Aldape K Barton MC. Inhibition of LSD1 sensitizes glioblastoma cells to histone deacetylase inhibitors. Neuro-Oncology. (2011) 13:894–903. doi: 10.1093/neuonc/nor049, PMID: 21653597 PMC3145466

[22] Ulrich S Ricken R Adli M. Tranylcypromine in mind (part I): review of pharmacology. Eur Neuropsychopharmacol. (2017) 27:697–713. doi: 10.1016/j.euroneuro.2017.05.007, PMID: 28655495

[23] Maes T Mascaro C Tirapu I Estiarte A Ciceri F Lunardi S. ORY-1001, a potent and selective covalent KDM1A inhibitor, for the treatment of acute leukemia. Cancer Cell. (2018) 33:495–511.e12. doi: 10.1016/j.ccell.2018.02.002, PMID: 29502954

[24] Hodges S Cooney J. Novel lysine-specific histone demethylase 1 inhibitor in acute myeloid leukaemia transformed from essential thrombocythaemia. Cancer Rep (Hoboken). (2021):e1588. doi: 10.1002/cnr2.1588 34786883 PMC9351673

[25] Antonijoan RM Ferrero-Cafiero JM Coimbra J Puntes M Martinez-Colomer J Arevalo MI. First-in-human randomized trial to assess safety, tolerability, pharmacokinetics and pharmacodynamics of the KDM1A inhibitor Vafidemstat. CNS Drugs. (2021) 35:331–44. doi: 10.1007/s40263-021-00797-x, PMID: 33755924 PMC7985749

[26] Bailey CP Figueroa M Gangadharan A Yang Y Romero MM Kennis BA. Pharmacologic inhibition of lysine-specific demethylase 1 as a therapeutic and immune-sensitization strategy in pediatric high-grade glioma. Neuro-Oncology. (2020) 22:1302–14. doi: 10.1093/neuonc/noaa058, PMID: 32166329 PMC7523459

[27] Lee C Rudneva VA Erkek S Zapatka M Chau LQ Tacheva-Grigorova SK. Lsd1 as a therapeutic target in Gfi1-activated medulloblastoma. Nat Commun. (2019) 10:332. doi: 10.1038/s41467-018-08269-5, PMID: 30659187 PMC6338772

[28] Wang Q Hu B Hu X Kim H Squatrito M Scarpace L. Tumor evolution of glioma-intrinsic gene expression subtypes associates with immunological changes in the microenvironment. Cancer Cell. (2017) 32:42–56.e6. doi: 10.1016/j.ccell.2017.06.003, PMID: 28697342 PMC5599156

[29] Phillips HS Kharbanda S Chen R Forrest WF Soriano RH Wu TD. Molecular subclasses of high-grade glioma predict prognosis, delineate a pattern of disease progression, and resemble stages in neurogenesis. Cancer Cell. (2006) 9:157–73. doi: 10.1016/j.ccr.2006.02.019, PMID: 16530701

[30] Bailey CP Figueroa M Gangadharan A Lee DA Chandra J. Scaffolding LSD1 inhibitors impair NK cell metabolism and cytotoxic function through depletion of glutathione. Front Immunol. (2020) 11:2196. doi: 10.3389/fimmu.2020.02196, PMID: 33042135 PMC7527493

[31] Singh MM Johnson B Venkatarayan A Flores ER Zhang J Su X. Preclinical activity of combined HDAC and KDM1A inhibition in glioblastoma. Neuro-Oncology. (2015) 17:1463–73. doi: 10.1093/neuonc/nov041, PMID: 25795306 PMC4648298

[32] Wang X Shi B Zhao Y Lu Q Fei X Lu C. HKDC1 promotes the tumorigenesis and glycolysis in lung adenocarcinoma via regulating AMPK/mTOR signaling pathway. Cancer Cell Int. (2020) 20:450. doi: 10.1186/s12935-020-01539-7, PMID: 32943998 PMC7488676

[33] Koss DJ Campesan S Giorgini F Outeiro TF. Dysfunction of RAB39B-mediated vesicular trafficking in Lewy body diseases. Mov Disord. (2021) 36:1744–58. doi: 10.1002/mds.28605, PMID: 33939203

[34] Schonberg DL Miller TE Wu Q Flavahan WA Das NK Hale JS. Preferential iron trafficking characterizes glioblastoma stem-like cells. Cancer Cell. (2015) 28:441–55. doi: 10.1016/j.ccell.2015.09.002, PMID: 26461092 PMC4646058

[35] Ravi V Madhankumar AB Abraham T Slagle-Webb B Connor JR. Liposomal delivery of ferritin heavy chain 1 (FTH1) siRNA in patient xenograft derived glioblastoma initiating cells suggests different sensitivities to radiation and distinct survival mechanisms. PLoS One. (2019) 14:e0221952. doi: 10.1371/journal.pone.0221952, PMID: 31491006 PMC6730865

[36] Oh CK Ha M Han ME Heo HJ Myung K Lee Y. FAM213A is linked to prognostic significance in acute myeloid leukemia through regulation of oxidative stress and myelopoiesis. Hematol Oncol. (2020) 38:381–9. doi: 10.1002/hon.2728, PMID: 32124993

[37] Faletti S Osti D Ceccacci E Richichi C Costanza B Nicosia L. LSD1-directed therapy affects glioblastoma tumorigenicity by deregulating the protective ATF4-dependent integrated stress response. Sci Transl Med. (2021) 13:eabf7036. doi: 10.1126/scitranslmed.abf7036 34878824

[38] Sacilotto N Dessanti P Lufino MMP Ortega A Rodríguez-Gimeno A Salas J. Comprehensive in Vitro Characterization of the LSD1 small molecule inhibitor class in oncology. ACS Pharmacol Transl Sci. (2021) 4:1818–34. doi: 10.1021/acsptsci.1c00223, PMID: 34927013 PMC8669716

[39] Shen H Laird PW. In epigenetic therapy, less is more. Cell Stem Cell. (2012) 10:353–4. doi: 10.1016/j.stem.2012.03.012 22482500

[40] Tsai H-C Li H Van Neste L Cai Y Robert C Rassool Feyruz V. Transient low doses of DNA-Demethylating agents exert durable antitumor effects on hematological and epithelial tumor cells. Cancer Cell. (2012) 21:430–46. doi: 10.1016/j.ccr.2011.12.029, PMID: 22439938 PMC3312044

[41] Salamero O Montesinos P Willekens C Pérez-Simón JA Pigneux A Récher C. First-in-human phase I study of Iadademstat (ORY-1001): a first-in-class lysine-specific histone demethylase 1A inhibitor, in relapsed or refractory acute myeloid leukemia. J Clin Oncol. (2020) 38:4260–73. doi: 10.1200/JCO.19.03250, PMID: 33052756 PMC7768337

[42] Cai SF Chu SH Goldberg AD Parvin S Koche RP Glass JL. Leukemia cell of origin influences apoptotic priming and sensitivity to LSD1 inhibition. Cancer Discov. (2020) 10:1500–13. doi: 10.1158/2159-8290.CD-19-1469, PMID: 32606137 PMC7584353

